# Pneumatic Differentiation and Medication

**Published:** 1887-01

**Authors:** H. A. Johnson

**Affiliations:** Emeritus Professor of Principles and Practice of Medicine in Chicago Medical College


PNEUMATIC DIFFERENTIATION AND MEDICATION. *By* H. A. JOHNSON, M.D., LL.D., *Emeritus Professor of Principles and Practice of Medicine in Chicago Medical College.*

[Read before the Chicago Medical Society, December 20th, 1886.]

The question of pneumatic differentiation has been quite largely discussed by members of the medical profession during the last two or three years, but there seems still to be a good deal of mystification on the subject. I was unable to be present when the matter was brought before this society. I therefore beg permission to say a few words which I had intended to say at that time and also to exhibit a contrivance for medication by spray or vapor in condensed air. It is not my purpose to discuss the merits of pneumatic differentiation. The subject, if not the term, has been before the profession for many years, and various devices have been employed for its accomplishment. The manufacturers of pneumatic cabinets insist that the desired results can be realized only by placing a patient in a box with a tube by means of which he breathes, the air of the room, while the pressure on the surface of the body is either diminished or increased by pumping air out of the box or into it. It is claimed that the result upon the body must be quite different from that reached by the use of the Waldenburg apparatus or other similar devices, for the reason that in some way the movement of a body under the pressure of a force, we will say, of fourteen pounds against a resistance of thirteen pounds, in which the available moving force is one pound, must be quite a different process from that which is reached when the moving force is fifteen pounds and the resisting force fourteen pounds. They do not, it is true, state it in this form, but they do assert that, in case we will say of the patient breathing through a tube the external air while the air in the chamber has been partly exhausted, so that its pressure is one pound per square inch less than the outside air, a *vis a fronte* is developed, by which the fluids and gases of the body are moved in a manner quite different from that which takes place when the patient sitting in the room breathes from a tank air compressed so that the pressure of the air breathed is one pound per square inch greater than that of the air in contact with the surface of the body. It must be evident that there is a fallacy in this claim.

We no longer use the phrase, *vis a fronte*, in the sense of an active force when we apply it to such phenomena as those which occur in the case of a vacuum filled by in-rushing matter. It is well-known now that there is an active force from behind, a *vis a tergo*, which pushes into a partial vacuum sufficient matter to equalize the force, whatever it be, on the other side, or to produce an equilibrium of force. In the pneumatic cabinets there is therefore only another mechanical device for affecting the differentiation produced by the Waldenburg apparatus, and which has repeatedly been produced by breathing air from a tank into which it has been condensed by some means, such as air pumps, water pressure, etc. I am not alone in holding this opinion.

Dr. Isaac Hull Platt in a paper read before the American Climatological Association at its third annual meeting, is led to conclude that the effects of breathing condensed air from the cabinet, the patient sitting in the room, are the same as those produced when the patient, placed in the cabinet and the air pressure reduced about the body, is allowed to breathe the air from the room. He says:

"To put the matter beyond a doubt," that is the claim of a special value in the inclosure of the patient in the cabinet, "I have reversed the breathing tube of the cabinet, placing the patient on the outside and compressed the air within the cabinet. The effects produced upon the residual air and upon the pulse, as well as the subjective experience of the person operated upon, were found to be identical with those obtained when he was within the cabinet and the pressure reduced to the same degree."

I have made quite a number of experiments bearing upon the same question with results in no sense differing from those reached by Dr. Platt. The proposition to conduct medicated sprays into the alveoli of the lungs by the differentiation of air pressure has been also ably treated by Dr. Platt, but I do not desire to consider it in this connection. I presume all admit that to the upper air passages sprays may be applied with, in many cases, benefit. The use of sprays or vapors with condensed air is conveniently accomplished by the use of the cabinet, but this result can be and has been repeatedly reached, and just as easily, by other devices. I have within the last twenty years resorted to several different contrivances for that purpose; an ordinary atomizing tube may be inserted through an opening in the tube from the tank of compressed air, so that medicinal substances are thrown in the form of spray into the stream of condensed air inhaled. There are quite a number of ways of accomplishing this: that which I have more recently used and which I submit to the society as a sample of what may be done, consists of a glass tube (I employ an ordinary percolator such as pharmacists use) to one end of which a breathing tube is attached and to the other end through a cork the atomizing tube and also the tube from my tank of condensed air. I at one time used a double tank, or rather two tanks, with an air gauge and stop cocks, so that I could maintain any required pressure in the tank from which my patient breathes. This tank may be a simple boiler, such as is used in kitchens for heating water for circulation through the house, say eighty gallons or more, or it may be in any other form desired. As the pressure is never great, not more usually than one-half or at most three-fourths of a pound to the square inch, it may be made of wood. A strong, tight cask or barrel even will answer the purpose. The ordinary form of pneumatic cabinet—the New York cabinet or the Pine cabinet—may be used as a tank, but it is unnecessarily heavy and clumsy and expensive. As I have a Pine cabinet in my office, I use it as a tank, with an 8 inch air pump for compressing the air. A copper or sheet iron tank that can be obtained of any plumber at a small fraction of the expense of the cabinet is quite as useful. Any physician who has a spray tube and glass vessel with two openings, a wolf bottle or even an ordinary wide-mouthed bottle, can provide himself with an apparatus just as useful as the pneumatic cabinet. By the use of a three way stop-cock expiration may be made into a tank of compressed or rarefied air, or against a valve supported by a spring of any desired pressure, or through a narrowed opening, so as to require force to expel the air from the chest. All these methods have been used and accomplish the same result, as expiration from the cabinet into outside air. The simpler the thing, provided it works, the better. The less mystery thrown around the whole subject, the better. I am quite confident that the physiological and therapeutical results obtained by the pneumatic cabinet are only such as may be reached equally well by the Waldenburg apparatus or by the still more simple means used some years since by the late Dr. Frank H. Davis, of this city. The apparatus is within the reach of anyone having a tank for condensed air for the purpose of atomizing or vaporizing medicinal substances, and requires no more skill or knowledge in its use than is required to administer narcotics, antipyretics or anæsthetics.

